# Two-dimensional assessment of submillimeter cancer-free margin area in colorectal liver metastases

**DOI:** 10.1097/MD.0000000000004080

**Published:** 2016-07-08

**Authors:** Takeshi Takamoto, Yasuhiko Sugawara, Takuya Hashimoto, Kei Shimada, Kazuto Inoue, Yoshikazu Maruyama, Masatoshi Makuuchi

**Affiliations:** Divisions of Hepato–Biliary–Pancreatic and Liver Transplantation Surgery, Japanese Red Cross Medical Center, Tokyo, Japan.

**Keywords:** colorectal liver metastasis, hepatectomy, liver, surgical margin

## Abstract

The aim of the study is to evaluate the prognostic impact of the extent of submillimeter or zero surgical margin (SubMM) area among the patients who underwent liver resection for colorectal liver metastases (CRLM).

The influence of suboptimal margin width of <1 mm on long-term outcome is unclear.

A total of 423 liver resections for CRLM were performed at Japanese Red Cross Medical Center between 2007 and 2015. Among them, we identified 235 patients who underwent curative initial liver resection and classified them into 2 groups: R0 (margin: ≥1 mm) and R1 (SubMM). The R1 group was further divided into 2 groups by the extent of SubMM area: small SubMM area (≤4 cm^2^) and broad SubMM area (>4 cm^2^).

The median tumor number was 4 (range 1–97), 23% had solitary and 37% had 8 or more number of metastases. With a median follow-up period of 30 months, the overall 1-, 3-, 5-year survival for R0 (n = 72) versus R1 (n = 163) groups were 98.4% vs 87.5%, 75.5% versus 57.1%, and 50.1% versus 36.6%, respectively (*P* = 0.004). After propensity score analysis allowing for matching the tumor number (<8 vs 8 or more), tumor size, and serum carcinoembryonic antigen level, the DFS and OS were significantly higher in the small SubMM area group (*P* = 0.024, *P* = 0.049), respectively.

Although wide margins >1 mm should be attempted whenever possible, reducing the extent of SubMM area (≤4 cm^2^) can contribute to better long-term outcome when wide margin is not practicable.

## 1 Introduction

Liver resection remains the best option for colorectal liver metastases (CRLM) and can provide long-term survival with 5-year survival rate of up to 40% in selected patients.^[1–3]^ Recent progress of modern chemotherapeutic agents and improved safety of liver resection contributes to expanding the indication of liver resection for CRLM patients with larger number and size of tumors.^[1,2]^ However, even though the number and size of metastatic liver tumors increases, it remains essential to preserve sufficient remnant liver volume and to remove tumors with sufficient surgical margin around the tumor simultaneously.

Recent observational studies demonstrated that one millimeter (1 mm) was enough as a minimum required cancer-free margin for CRLM, showing that patients with 1 mm margin had similar long-term survival to those with one centimeter (1 cm) margin.^[4,5]^ These studies, however, evaluated surgical margin 1-dimensionally, and it is unclear whether the extent of suboptimal (<1 mm or zero) surgical margin area influences on the long-term outcome after liver resection.

In this study, we estimated the area of submillimeter or zero surgical margin (SubMM) before and after slicing resected specimens for pathological examination. We aimed to assess whether liver resection with small SubMM area can provide better a long-term outcome than that with broad SubMM area.

## 2 Methods

### 2.1 Patients

A total of 1341 liver resection for all diagnosis were performed at Japanese Red Cross Medical Center between 2007 and 2015. Among them, 423 patients underwent liver resection for CRLM. Those who had any of the following conditions were excluded from the study: (1) repeat hepatectomy (n = 183), (2) R2 resection (noncurative resection with macroscopically positive for residual cancer, n = 5). Therefore, clinical data, operative details, and pathological diagnosis of 235 patients were obtained from prospectively maintained database and analyzed. The study was approved by the Institutional Review Board of Japanese Red Cross Medical Center.

### 2.2 Surgical procedure

Our eligible criteria for liver resection were: (1) no unresectable extrahepatic disease was found in preoperative computed tomography (CT) scan, (2) sufficient remnant liver volume, which was determined by the individual result of indocyanine green retention test,^[6–8]^ can be expected. From 2008, the procedure of the liver resection was simulated^[9]^ and the future remnant liver volume was calculated preoperatively by 3-dimensional computer software (Synapse 3D, Fujifilm, Tokyo, Japan). Limited resections or anatomical segmentectomy were selected rather than major hepatectomy, if sufficient surgical margin was expected. Preoperative portal vein embolization was performed prior to liver resection, if necessary.^[10]^ Irrespective of the number of liver metastases, we indicated liver resection when patients met these criteria. As for operative procedure, liver parenchymal dissection was always performed by the Kelly clamp crush technique under intermittent Pringle maneuver. No energy devices were used except for electric cautery. We have used the Kelly clamp crush technique mainly in all liver resection from 1980 s.^[6]^ It can provide sensory feedback, especially when the Kelly tip comes very near to a tumor, which enables limited resection with minimum surgical margin. There were no patients who underwent a combination of resection and ablation or 2-staged hepatectomy. Postoperative complication was evaluated according to Clavien–Dindo's classification.^[11]^ After surgery, a postoperative follow-up program including contrast-enhanced CT scan and blood test including measurement of serum carcinoembryonic antigen (CEA) level every 2 to 3 months was applied for all patients.

### 2.3 Pathological analysis

Both surgeons and pathologists participated in the evaluation of surgical margin in specimens. Surgeons examined specimens by observation and palpation, and then sliced them into around 1-cm thick sections, including a slice of maximum diameter of the tumor and the narrowest surgical margin on liver transection surface. In addition to the number and size of liver tumors, the distance of narrowest margin from the tumor to the liver transection line was measured in the specimen. According to this 1-dimensional evaluation of surgical margin, the distance of narrowest surgical margin was classified into tumor exposure (zero margin), 0 to 1 mm (submillimeter) and >1 mm (more than 1 mm). Patients were divided into 2 groups by the distance of the narrowest surgical margin: a >1 mm surgical margin (R0) group and a zero margin and 0 to 1 mm margin (R1) group. In concordance with previous reports^[12–14]^ on surgical margin of CRLM assuming that the presence of microscopic residual tumor is highly suspected in patients with 1 mm or smaller surgical margin, patients with 1 mm surgical margin were categorized into the R1 group in this study.

Specimens with zero margin or submillimeter margin (SubMM) were sent to 2-dimensional evaluation for measuring the extent of SubMM area and were categorized into 4 groups: smaller than 1 cm^2^ (<1 × 1 cm^2^), 1 to 4 (= 1 × 1 – 2 × 2) cm^2^, 4 to 9 (= 2 × 2 – 3 × 3 cm^2^ and broader than 9 (>3 × 3) cm^2^. In actual procedure, when surgeons found suspicious area of SubMM, a fragment of 1-mm square paper was used to approximate the extent of SubMM area. The fragment papers were put on the suspicious area of SubMM, and the number of the papers to cover the SubMM area was counted. This approximation was possible by reassembling and building up the sliced specimens even if the SubMM area was found after slicing. Pathologists examined the specimens microscopically by haematoxylin and eosin staining and other staining. The minimum distance of surgical margin was recorded and the SubMM area was marked on gross photographs of specimens. If there was a discrepancy of macroscopic and microscopic assessment of SubMM area, macroscopic and microscopic assessment were reviewed and discussed by liver surgeons and pathologists. All the assessment of SubMM was supervised by a liver surgeon (T.T) by reviewing the reports and gross photographs of specimens, which were taken before and after slicing and formalin fixation.

### 2.4 Propensity score matching analysis

Patients in the R1 group were subdivided into 2 groups according to the extent of SubMM area: 4 cm^2^ or smaller (small SubMM group) and broader than 4 cm^2^ (broad SubMM group). Patients’ characteristics and extent of disease were compared between the patients with broad and small of SubMM area. Generally, when the number and size of tumor increase, the narrow surgical margin area tend to be broader. These factors are also known as surrogate prognostic factors for CRLM and can be confounding factors in assessing postoperative long-term outcome between the 2 groups. Propensity score matching was used to control these possible confounding factors.

### 2.5 Data analysis

Statistical analyses were carried out using JMP^®^ 11 (SAS Institute Inc., Cary, NC). Comparison between groups was made using the Mann–Whitney *U* test for continuous variables and using chi-square test or Fischer's exact test for categorical variables. Survival was analyzed using the Kaplan–Meier method. The log-rank test was used for comparison of survival between groups. All variables associated with survival with *P* < 0.2 in the univariate proportional hazard models were subsequently entered into a Cox multivariate regression models. *P* < 0.05 values were considered statistically significant. Propensity score matching was performed as one-to-one matching with caliper width of 0.10.

## 3 Results

### 3.1 Demographics and operative details

During the study period, 235 patients were eligible. There were no patients whose data was missing. The median age was 63 years (range 30–89) and 43% were females (Table 1). The median tumor number was 4(range 1–97), 23% had solitary metastasis, and the median diameter of largest tumor was 3.7 cm (range 0.7–20). In total, 64% had synchronous disease; 115 (49%) patients received preoperative chemotherapy including oxaliplatin and/or irinotecan. The median operation time was 448 minutes (120–1252) and blood loss was 775 mL (20–6200). The red blood cell transfusion rate was 11%. Extrahepatic lesions were concomitantly resected in 17%. According to macroscopic and microscopic pathological assessment, 72 (31%) patients had surgical margin with >1 mm and 163 (69%) had minimum surgical margin <1 mm (submillimeter margin) or tumor exposure on liver transection surface (zero margin). Postoperative complication was observed in 39% and morbidity of grade III or IV was observed in 13 (6%) patients: intra-abdominal abscess (n = 5) bleeding (n = 3), costal nerve pain requiring regional nerve blockade (n = 1), symptomatic pleural effusion (n = 1), pneumothorax (n = 1), wound dehiscence (n = 1), and paraplegia (n = 1). There was no postoperative liver failure and operation related 90-days mortality except for 1 patient who died 66 days after surgery for aggressive systemic recurrence. The median follow-up period was 30.0 months (interquartile range was 12.6–49.2 months) after liver resection.

**Table 1:**
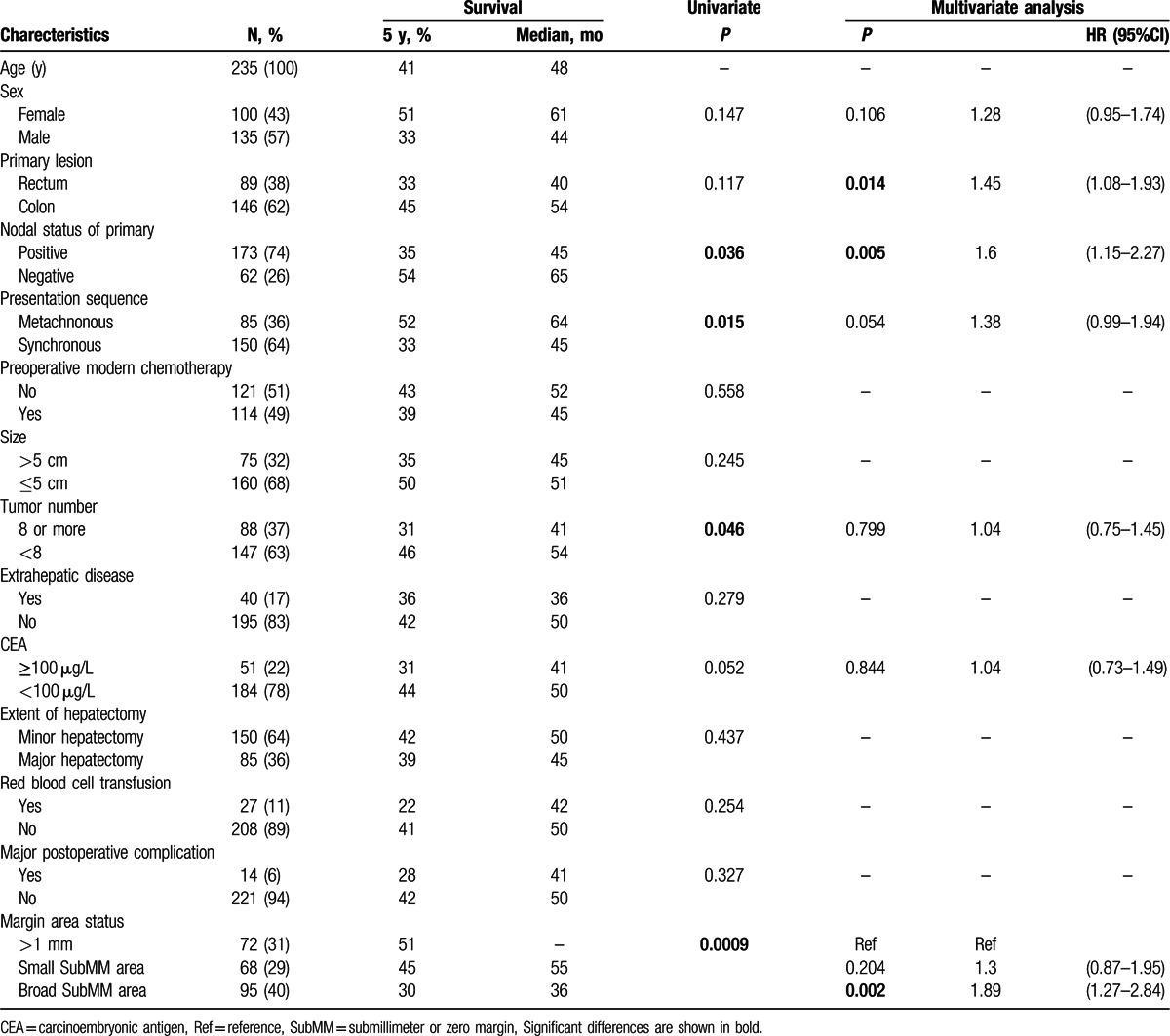
Characteristics of 235 patients with resected colorectal liver metastases and univariate/multivariate analyses of factors associated with overall survival.

### 3.2 Survival and surgical margin analysis

The overall 1-, 3-, 5-year survival for this cohort was 91%, 63%, 40.1%, respectively, and median survival was 48.2 months (95% CI: 42.0–56.1). The 1-, 3-, 5-disease-free survival (DFS) ratio was 29%, 12%, 10%, respectively, and the median DFS was 6.0 months (95% CI: 4.4–7.7).

**Figure 1:**
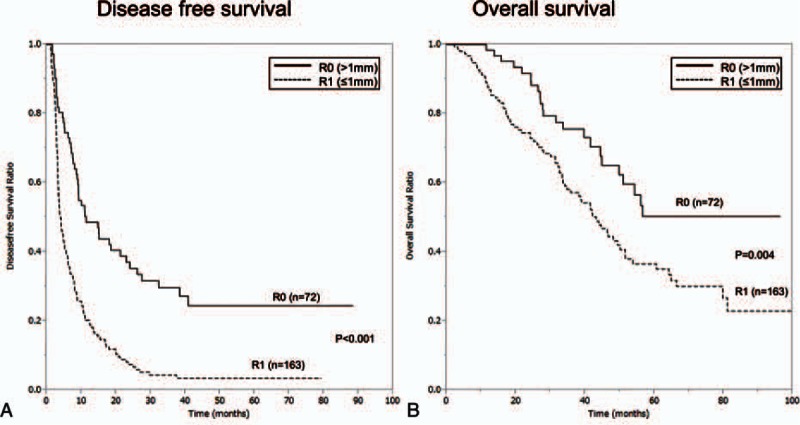
Survival comparisons (A, disease-free survival and B, overall survival) between R0 (0.1 mm surgical margin) and R1 (submillimeter or zero surgical margin) groups.

As a result of 1-dimensional assessment of surgical margin, 72 patients had >1 mm surgical margin and were categorized into R0, whereas 163 patients into R1. There was significant difference in DFS (*P* < 0.001) and overall survival (OS) (*P* = 0.004) between R0 and R1 groups (Fig. 1). The overall 1-, 3-, 5-year survival for R0 (>1 mm) versus R1 (SubMM) groups was 98.4% versus 87.5%, 75.5% versus 57.1%, 50.1% versus 36.6% and the DFS ratio at 1-,3-,5-year was 48.6% versus 20.2%, 29.7% versus 4.3%, 24.5% versus 3.5%, respectively.

### 3.3 Prognostic factors of OS

Results of analysis of predictors of OS in the eligible 235 patients were shown in Table 1. Univariate analysis identified 4 factors of predicting worse OS; positive nodal status of primary lesion (*P* = 0.036), synchronous (*P* = 0.015), 8 or more tumor number (*P* = 0.046), and broad area of SubMM area (*P* = 0.009). On multivariate analysis, positive nodal status of primary lesion (HR, 1.6; 95% CI, 1.15–2.27; *P* = 0.005) and broad SubMM area (HR, 1.89; 95% CI, 1.27–2.84; *P* = 0.002) were remained as significant predictors of worse OS.

### 3.4 Long-term outcome between small and broad area of SubMM groups

The surgical margin area of 163 patients with SubMM was evaluated and was categorized into 4 groups: <1 cm^2^ (n = 33), 1 to 4 cm^2^ (n = 35), 4 to 9 cm^2^ (n = 31), and broader than 9 cm^2^ (n = 64). The 3-year overall survival for each group was 68.1%, 66.8%, 46.0%, and 51%, respectively, and the 5-year overall survival for each group was 47.0%, 42.8%, 33.1%, and 28.3%, respectively (*P* = 0.214). Further analysis was performed by subdividing them into 2 groups; smaller than 4 cm^2^ (small SubMM area group; n = 68) and broader than 4 cm^2^ (broad SubMM area group; n = 95).

**Figure 2:**
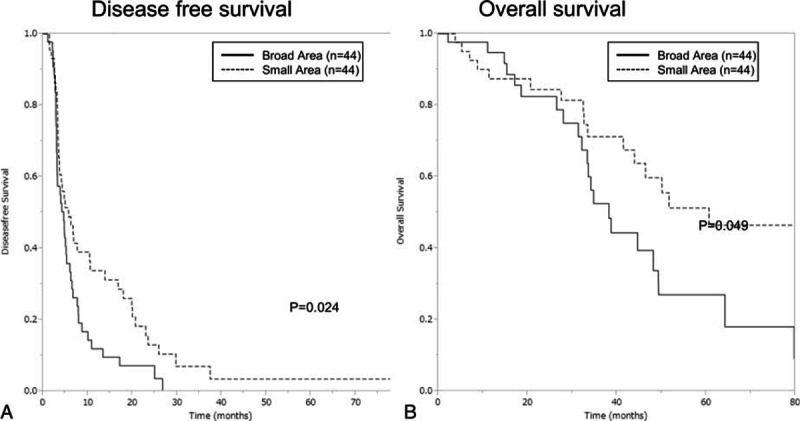
Survival comparisons (A, disease-free survival and B, overall survival) between small and broad submillimeter or zero surgical margin groups after propensity score matching.

As shown in Table 2, the patients in small SubMM area group had higher level of serum CEA, larger number and size of tumor than those in the broad SubMM area group (*P* < 0.001). Therefore, these 3 factors, the tumor number (8 or more), tumor size, and serum CEA value, were used for propensity score estimation. After propensity score matching, further analysis for long-term outcome was carried out. The patients with broad SubMM area were associated with larger amount of intraoperative blood loss (*P* = 0.043) and longer operation time (*P* = 0.012). The broad area groups had significantly shorter DFS and OS than the small area groups. The 1-, 3-, and 5- year DFS rates of the small area group were 34%, 7%, and 3% versus 12%, 4%, and 0% in the broad area group, respectively (*P* = 0.024). And the 1-, 3-, and 5-year OS rates of the small area group were 84%, 68%, and 42% versus 90%, 53%, and 26% in the broad area group, respectively (*P* = 0.041) (Fig. 2).

**Table 2:**
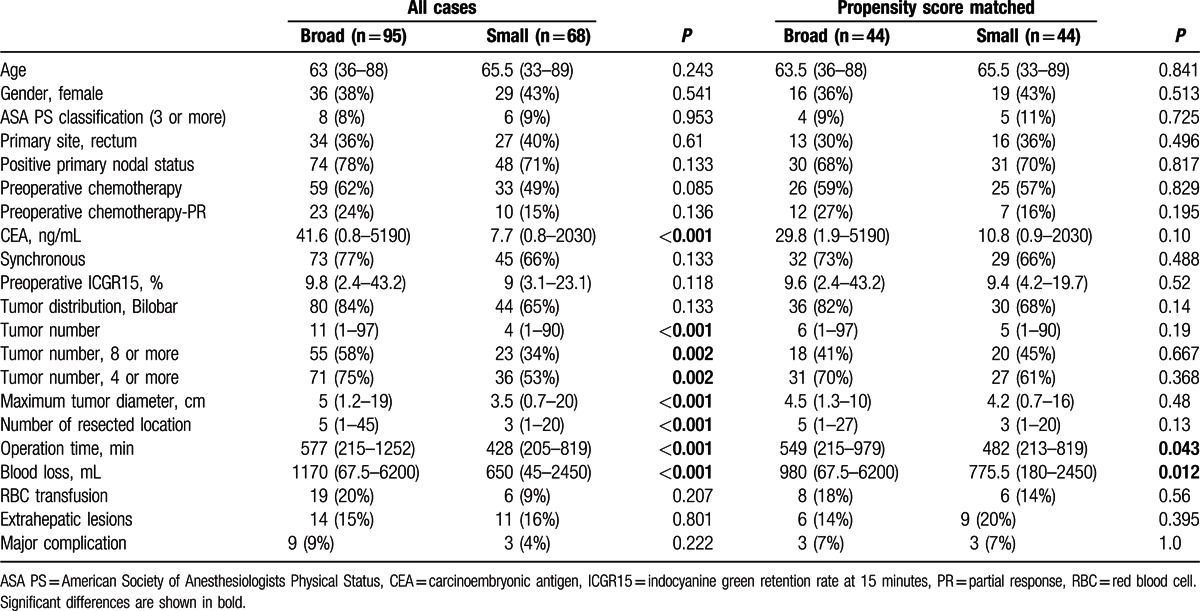
Characteristics of patients with broad and small area of submillimeter /zero surgical margin before and after propensity score matching.

## 4 Discussion

In this study, 235 patients who underwent initial curative resection for CRLM in a single institution were investigated. A wide surgical margin >1 mm was confirmed to be associated with better long-term outcome than SubMM. After propensity score matching, patients with small area (<4 cm^2^) of SubMM showed better DFS and OS than those with broad area.

There was a long-accepted consensus that a 1-cm cancer-free margin was necessary for curative resection of CRLM.^[15–18]^ It is also supported by microsatellite theory,^[19,20]^ insisting that 95% of microsatellite lesions when present were found within 1 cm from the tumor surface. At any time, sufficient surgical margin should be obtained to prevent tumor exposure. Nevertheless, recent progress in chemotherapy and consequent expansion of operative indication of CRLM advocate revision of this “1 cm margin consensus” on the basis of the following 2 major factors. First, sequential limited liver resections, which is called as “parenchymal sparing hepatectomy” (PSH), is now becoming a standard procedure^[2,21]^ and 1-cm surgical margin now cannot be regarded as a practical standard in performing PSH. PSH was shown to offer more opportunities for repeat hepatectomy without increasing hepatic recurrence^[3]^ with similar or even better long-term outcome^[22–25]^ than major hepatectomy. Additionally, the emergence of chemotherapy-induced liver injury^[8,26,27]^ evokes the necessity of liver parenchymal sparing.^[27,28]^ However, when the tumor number and size increase, it becomes technically more demanding and difficult to keep sufficient margin through the wide and time-consuming operative procedure of limited resections avoiding major vascular injury. The possibility of tumor exposure on liver transection line raises, as tumor shape is not always round and smooth. Second, 1-cm surgical margin can be too large to remain sufficient remnant liver volume especially when the tumor number increases. Mathematically, the volume of normal liver parenchyma surrounding a tumor, if the tumor assumed as a sphere of 1 to 5 cm in diameter, amounts to 3.7 to 47.6 mL when surgical margin is settled as 1 cm, whereas it amounts to 0.2 to 4 mL when surgical margin is settled as 1 mm. The total volume of concomitantly resected normal liver parenchyma as surgical margin will be obtained by multiplying these values by the number of tumor or resection site. And if portal branches and hepatic venous tributaries are involved in the surgical margin, their peripheral territories will lose normal function. Therefore, sequential limited resections with plenty surgical margin around tumors can remain considerably small functional liver volume in some cases. Hamady et al showed that similar OS can be expected for patients with 1 to 10 mm margin as those with >10 mm margin.^[4,5]^ They also demonstrated that worse OS in patients with submillimeter margin than those with wider margin. Consequently, 1 mm is now regarded as the acceptable minimum limit of surgical margin for CRLM.

The most noteworthy findings in the present study is that patients with small area (<4 cm^2^) of SubMM had significantly longer DFS and OS than those with broad area after propensity score matching. This result indicates that it can contribute long-term outcome to make the SubMM area smaller than about a finger tip unit area, even when wider surgical margin cannot be obtained. On the contrary, the broad SubMM area was associated with worse survival even after eliminating suspicious confounding factors by propensity score matching. From the oncological point of view, tumor expose or spreading during liver resection can induce recurrence of peritoneal dissemination and local recurrence around liver transection surface, which is difficult to diagnose and to treat surgically. Liver resection with broad subMM area has a possibility to reduce the chance of additional surgical treatment for disease recurrence. Preoperative 3-dimensional simulation analysis is helpful in planning several operative procedures and choosing an optimal procedure, which has sufficient surgical margin between liver transecting line and tumor surface.^[9,29]^ However, there are other technical difficulties in performing liver resection as planned. It is essential for liver surgeons to follow the liver transection line constantly during the liver transection in order to keep sufficient margin between the line and tumor surface using intraoperative ultrasonography (IOUS) and contrast-enhanced IOUS when the tumor border is not clearly visualized.

As a nature of retrospective study, this analysis has inherent selection limitations. As the possibility of broad SubMM area raises as the tumor number increases, SubMM can be a confounding factor of larger number of CRLM, which is a widely-accepted risk factor of poor prognosis. In this study, a relatively large number of patients had 8 or more tumor number (37%) and resectable extrahepatic lesions (17%), compared to previous studies of surgical treatments for CRLM.^[4,5,14,21]^ This difference of study population may affect these widely accepted poor prognostic factors had less impact on survival than margin area status. It is also well known that prognosis of CRLM patients is affected by multiple factors including treatment strategies for recurrence and initial treatment may play little role for them. However, the present study was designed to eliminate these confounding factors and selected patients who underwent initial surgical treatment for CRLM by the same decision-making criteria for operative procedure, surgical technique of liver resection, pathological assessment, and postoperative treatment including repeat resection for recurrence in a single institution. In addition, the study period starts from 2007, when all the modern chemotherapeutic agents are available for all patients in our country.

In conclusion, this study provides evidence about the 2-dimensional assessment of SubMM area and that the small SubMM area for CRLM is associated with better OS and DFS than the broad SubMM area. The broad SubMM area is an independent predictor of OS. These findings emphasize the importance of constant intraoperative monitoring of liver transection line to keep sufficient surgical margin from the tumor border.
